# Biogenic Amine Production by Lactic Acid Bacteria: A Review

**DOI:** 10.3390/foods8010017

**Published:** 2019-01-07

**Authors:** Federica Barbieri, Chiara Montanari, Fausto Gardini, Giulia Tabanelli

**Affiliations:** 1 Interdepartmental Center for Industrial Agri-Food Research, University of Bologna, 47521 Cesena, Italy; federica.barbieri16@unibo.it (F.B.); chiara.montanari8@unibo.it (C.M.); fausto.gardini@unibo.it (F.G.); 2 Department of Agricultural and Food Sciences, University of Bologna, 40126 Bologna, Italy

**Keywords:** biogenic amines, decarboxylase enzymes, lactic acid bacteria, starter cultures

## Abstract

Lactic acid bacteria (LAB) are considered as the main biogenic amine (BA) producers in fermented foods. These compounds derive from amino acid decarboxylation through microbial activities and can cause toxic effects on humans, with symptoms (headache, heart palpitations, vomiting, diarrhea) depending also on individual sensitivity. Many studies have focused on the aminobiogenic potential of LAB associated with fermented foods, taking into consideration the conditions affecting BA accumulation and enzymes/genes involved in the biosynthetic mechanisms. This review describes in detail the different LAB (used as starter cultures to improve technological and sensorial properties, as well as those naturally occurring during ripening or in spontaneous fermentations) able to produce BAs in model or in real systems. The groups considered were enterococci, lactobacilli, streptococci, lactococci, pediococci, oenococci and, as minor producers, LAB belonging to *Leuconostoc* and *Weissella* genus. A deeper knowledge of this issue is important because decarboxylase activities are often related to strains rather than to species or genera. Moreover, this information can help to improve the selection of strains for further applications as starter or bioprotective cultures, in order to obtain high quality foods with reduced BA content.

## 1. Biogenic Amine Toxicity and Physiological Role in Microorganisms

A large number of metabolites, exerting both beneficial and detrimental properties for human health, can be synthetized by microorganisms. Among these, amino acid derivatives produced during bacterial growth and fermentation can interact with human physiology in several ways, showing health-modulating potential [1]. This group includes bioactive compounds such as biogenic amines (BAs), which are responsible for adverse effects and are involved in several pathogenic syndromes [1]. In fact, ingestion of food containing high BA amounts is a risk for consumer health since these compounds can cause headache, heart palpitations, vomiting, diarrhea and hypertensive crises [2,3,4]. However, their toxic effect depends on the type of BA, on individual sensitivity or allergy and on the consumption of monoaminooxidase inhibitory drugs or ethanol, which interact with aminooxidase enzymatic systems responsible for the detoxification process of exogenous BAs [5,6].

Due to the severity of symptoms they may cause, histamine and tyramine are the most dangerous BAs and are responsible for symptomatology known as “scombroid fish poisoning” and “cheese reaction,” respectively [3,7]. The “scombroid fish poisoning”, often due to the consumption of fish such as tuna, sardines, anchovies, mackerel, etc., consists in flushing of face, neck and upper arms, oral numbness and/or burning, headache, heart palpitations, asthma attacks, hives, gastrointestinal symptoms, and difficulties in swallowing [8]. Tyramine intoxication is known as “cheese reaction” because this BA is the most frequently found in cheese and it can causes dietary-induced migraine, increased cardiac output, nausea, vomiting, respiratory disorders and elevated blood glucose [7,9]. As far as other BAs, the presence of high level of 2-phenylethylamine, putrescine, cadaverine, agmatine, spermine and spermidine can lead to toxicity. Moreover, they can potentiate the effects of histamine and tyramine toxicity by inhibiting their metabolizing enzymes [10].

Although the consumption of food containing large amounts of BAs can have toxicological consequences, there is no specific legislation regarding the presence of BAs in foods, with the exception of fishery products, for which the maximum acceptable level of histamine is defined [11]. However, recently, EFSA conducted a qualitative risk assessment concerning BA in fermented foods in the European Union, indicating concentrations that could induce adverse effects in consumers [12].

According to their chemical structures, BAs can be classified as aromatic (tyramine and 2-phenylethylamine), aliphatic (putrescine, cadaverine, spermine and spermidine) and heterocyclic (histamine and tryptamine) (see Table 1) and they are analogous to those naturally found in fresh food products, which exert a physiological role associated with cell growth and proliferation [13,14].

The exogenous BAs derive from bacterial decarboxylation of the corresponding amino acids through decarboxylase enzymes. Histamine and cadaverine can be formed by converting histidine and lysine via histidine decarboxylase (HDC) and via lysine decarboxylase (LDC), respectively. Tyrosine is converted in tyramine by tyrosine decarboxylase (TDC), which can act also on phenylalanine obtaining 2-phenylethylamine. This latter aromatic BA is produced by TDC with a lower efficiency with respect to tyramine and it is accumulated when tyrosine is almost completely depleted [15,16,17]. The formation of these BAs is based on one-step decarboxylation reactions of their respective amino acids and requires systems for amino acid active transport such as antiporter protein in exchange for the resulting BA. Putrescine can be accumulated with a single-step decarboxylation pathway by ornithine decarboxylase (ODC), common in Gram negative bacteria (such as enterobacteria and pseudomonads) or Lactic Acid Bacteria (LAB) deriving from wine environment [16,18,19,20]. However, this BA can also be formed through agmatinase pathway, which directly converts agmatine to urea and putrescine, or by agmatine deiminase (AgDI) pathway, common in LAB, which transforms arginine to agmatine by arginine decarboxylase. Subsequently, agmatine is converted to putrescine by the agmatine deiminase system, consisting of three enzymes: agmatine deiminase, putrescine carbamoyltransferase and carbamate kinase [21]. The biosynthesis of higher polyamines (spermine and spermidine) proceeds with complex pathways starting from putrescine released from ornithine or agmatine [22,23].

The decarboxylative pathways are activated for several physiological reasons. In fact, decarboxylation of amino acids is coupled with an electrogenic antiport system that can counteract intracellular acidification [24,25]. Therefore, BA accumulation can represent a cellular defense mechanism to withstand acid stress and it has been demonstrated that the transcription of many decarboxylase genes is induced by low pH and improves cell performances in acid conditions [9,17,19,20,26,27]. Moreover, the transfer of a net positive charge outside the cell can generate a proton motive force, leading to cell membrane energization and bringing supplementary energy. It has been demonstrated that the decarboxylase pathway can support the primary metabolism in environmental critical conditions [26,27,28]. This function can be particularly important for microorganisms lacking a respiratory chain, such as most LAB [29]. del Rio et al. [30] demonstrated that AgDI pathway promotes the growth of *Lactococcus lactis* after nutrient depletion.

Several Gram negative and Gram positive bacteria are able to produce BAs. Spoilage bacteria belonging to enterobacteria and pseudomonads can accumulate histamine, putrescine and cadaverine [47,64,73,74,75]. For this reason, BA content has been related to poor hygienic quality of non-fermented foods, being associated with a massive growth of decarboxylase positive spoilage microorganisms, and several authors proposed BA content as a microbial quality index [75,76]. Decarboxylase activity has been described also in Gram positive microbial groups, such as staphylococci, *Bacillus* spp. and, especially, LAB, considered the most efficient tyramine producers [9,48]. Moreover, the ability to produce histamine, cadaverine and putrescine by bacteria belonging to LAB have been reported [21,64,77]. According to some authors, also yeasts and moulds are implicated in BA accumulation, even if with a controversial role [78,79,80]. It is important to point out that the capability to produce BAs is generally a strain-specific characteristic, with strong variability in aminobiogenetic potential between different strains belonging to the same species.

## 2. Role of LAB in Fermented Food BA Content and Their Decarboxylase Clusters Genetic Organization

BA content in fermented foods is of great interest not only for its potential health concerns but also from an economic point of view. On the other hand, the presence of small concentrations of these compounds in fermented foods is unavoidable. In fact, the BA content in these products can range from concentrations below 20 mg/kg for alcoholic and no-alcoholic beverages, fermented vegetables and soy products, up to several hundred mg/kg for some sausages and cheeses [12]. The presence of different BAs is dependent on the precursor availability due to proteolysis during ripening. Moreover, the presence of decarboxylase positive non-starter microbiota, deriving from raw material and productive environment, often leads to high BA concentrations in fermented foods, especially in those obtained without the use of starter cultures [47,75,81,82]. In addition to precursor availability and the presence of BA producing microorganisms, the accumulation of these compounds depends on various intrinsic, environmental and technological factors, recently revised by Gardini et al. [83].

Decarboxylase activity is often expressed independently of cell viability and these enzymes maintain their activity after cell lysis also in harsh environmental conditions [31,49,84,85]. Moreover, once produced, BAs are stable to heat treatment, freezing, and smoking [86].

Dairy products, especially ripened cheeses, have been associated with foodborne intoxications due to their high content of BAs, such as tyramine, histamine, putrescine, and 2-phenylethylamine [32,47]. In any case, BA content varies between different types of cheeses and even among different sections of the same cheese [87]. In fermented meats the most prevalent BAs are tyramine, cadaverine, putrescine, and, with minor extent, histamine and their levels strongly vary among different types of products and [75,88,89,90]. The presence of these compounds in such products depends on low quality processing conditions favoring contamination and on the presence of autochthonous microbiota with decarboxylase potential [82]. Also in alcoholic beverages BAs (mainly histamine, tyramine, putrescine and cadaverine) can be formed through microbial activity during production and storage [60,61]. The presence of BAs has been reported also in fermented vegetables, such as sauerkraut or table olives, where the presence of aminobiogenic spoilage microorganisms can result in high putrescine, cadaverine and tyramine content [91,92]. Abundant amounts of histamine have been detected in fermented fish products [93].

Even if the production of diamines is usually attributed to spoiling Gram negative bacteria, such as enterobacteria and pseudomonads [82], LAB are considered mainly responsible for BA production in fermented foods [47,94]. Although starter cultures are accurately selected for the absence of decarboxylase activity, non-controlled autochthonous LAB involved in ripening process can contribute to BA accumulation. These non-starter LAB (NSLAB) consist mainly of mesophilic facultative or obligate heterofermentative bacteria, which exert a crucial role in maturation phenomena such as the development of flavor [95,96]. These bacteria show good adaptation to unfavorable growth conditions and can survive for long period after sugar depletion, thanks to their ability to obtain energy for growth and survival from other substrates, among which amino acids [97,98,99,100]. Moreover, the adaptation to some ecological niches has required the capability to resist acid stresses, activating bacterial mechanisms able to counteract low pH. In stress conditions occurring in fermented foods during ripening, NSLAB encode specific genetic mechanisms that lead to stress responses producing physiological changes among which decarboxylation reactions acquire important roles thanks to the maintenance of pH homeostasis [24,25,101,102]. In fact, expression (by transcriptional induction) and/or activation (by catalytic modulation) of amino acid decarboxylation systems in LAB are reported to be adaptive responses to energy depletion but also strategies to counteract acid stress [103]. The presence of the decarboxylase genes involved in the production of BAs are mostly strain dependent rather than species specific, highlighting the occurrence of horizontal gene transfer between strains as part of a mechanism of survival and adaptation to specific environments [16,33,50]. Recently, the genes belonging to BA biosynthetic pathways in LAB have been identified and the genetic organization of decarboxylase clusters has been reviewed [9,34,64,65]. Generally, enzymes responsible for specific amino acid decarboxylation are organized in clusters in which some genes are always present, i.e., the specific amino acid decarboxylase and the corresponding antiporter permease.

The first tyrosine decarboxylase locus (*tdc*) described in bacteria was found in *Enterococcus faecalis* JH2-2 [104]. This cluster has been annotated also in the genome sequence of other LAB [16,51,52,105,106,107]. Marcobal et al. [9] evidenced for all tyramine biosynthetic loci a high similarity in both gene sequence and organization, since this locus usually contains the genes encoding tyrosine decarboxylase (*tyrDC*), tyrosyl tRNA synthetase (*tyrS*, located upstream the *tyrDC* gene), putative tyrosine/tyramine permease (*tyrP*, located downstream the *tyrDC* gene) and a Na^+^/H^+^ antiporter (*nhaC*) [47]. The similar organization of different *tdc* clusters, their distribution, and their high similarity of sequence suggest a horizontal transfer of this cluster from a common source [106]. However, different strains can have different transcriptional organizations of the *tdc* gene cluster, as demonstrated by reverse transcription polymerase chain reaction (PCR) analyses. In fact, the four complete Open Reading Frame (ORF) can be co-transcribed [53] or *tyrS* can be transcribed independently and not included in the catabolic operon [27].

The LAB histidine decarboxylases belong to pyruvoil-dependent decarboxylases group and the encoding histidine decarboxylase gene (*hdcA*) has been identified in several LAB species [33,34,35,85,108,109,110,111,112,113]. The histidine decarboxylase gene clusters (*hdc*) of Gram positive bacteria usually comprise the decarboxylase gene *hdcA* and the histidine/histamine antiporter gene *hdcP*. Frequently, an *hdcB* gene, involved in the conversion of the histidine decarboxylase proenzyme to the active decarboxylase can be found [114]. Moreover, for lactobacilli, a histidyl-tRNA synthetase (*hisS*) gene has also been described [35]. The transcriptional studies demonstrated that these genes are located on an operon transcribed as a polycistronic mRNA. However, some authors demonstrated that the antiporter gene is transcribed as a monocistronic RNA and that transcriptional termination structures are present in the intergenic regions of histamine operon in *Lactobacillus buchneri* [111]. Rossi et al. [85] found that *hdcA* gene of *Streptococcus thermophilus* PRI60 was genetically different from the *hdcA* genes sequenced in other LAB, in agreement with the findings of Calles-Enríquez et al. [35], who reported that *hdc* cluster of *S. thermophilus* was more closely related to genera such as *Clostridium* and *Staphylococcus* than other LAB. Another interesting feature of *hdc* gene is its possibility to be located on a plasmid [34]. Lucas et al. [33,36] found that *Lactobacillus hilgardii* 0006, *Tetragenococcus muriaticus*, and *Oenococcus oeni* strains showed 99 to 100% identical *hdcA*- and *hdcB*-encoded proteins, highlighting the presence of a plasmid-encoded histidine decarboxylase system recently transferred horizontally between bacteria. Furthermore, they found that the *hdc* gene cluster, responsible for histamine production in *L. hilgardii* IOEB 0006, was located on an 80-kb plasmid that proved to be unstable. In fact, the capability to form histamine was lost in relation to the growth conditions.

Depending on the producer bacterium, genes/enzymes involved and the ecological niche from which it originates, two different metabolic routes have been described in LAB for the biosynthesis of putrescine [20,64,115]. The first is a decarboxylation system consisting of an ornithine decarboxylase (ODC) and an ornithine/putrescine exchanger. These enzymes are encoded by a gene cluster containing two adjacent genes: (i) *speC* encoding a biosynthetic/constitutive form of the ODC enzyme and (ii) *potE* encoding the transmembrane substrate/product exchanger protein [19,20,116]. Gram positive bacteria, however, have been infrequently reported to possess an ODC enzyme and putrescine-producing LAB strains via the ODC pathway are essentially, although not exclusively, derived from wine environment, belonging to the species *Lactobacillus saerimneri*, *Lactobacillus brevis* [19,20], *Lactobacillus mali* [18], and *O. oeni* [66]. In contrast, the agmatine deiminase (AgDI) pathway is relatively frequent in LAB and it is even considered a species trait in some enterococci [54]. This pathway consists of a more complex system, comprising AgDI, a putrescine transcarbamylase, a carbamate kinase, and an agmatine/putrescine exchanger [65,101]. Five genes are grouped in the agmatine deiminase cluster (*AgDI*): the regulator gene *aguR* and the metabolic genes *aguB*, *aguD*, *aguA* and *aguC* (*aguBDAC*). Linares et al. [117] reported that *aguR* is constitutively transcribed from its promoter (P*aguR*) while the catabolic genes are co-transcribed in a single mRNA from the *aguB* promoter (P*aguB*) in a divergent orientation. These pathway genes were occasionally detected in a putative acid resistance locus in LAB species [101]. In this locus, the *AgDI* genes are found adjacent to the genes associated with the tyrosine decarboxylase pathway on the chromosome [53], suggesting the presence of genes for high-alkalinizing routes (such as amino acid decarboxylases) in LAB genome.

## 3. Main LAB Involved in BA Production in Fermented Foods

All fermented foods are subjected to the risk of BA contamination. Although LAB are considered GRAS (Generally Regarded As Safe) organisms, they can have the capability to produce toxic compounds as BAs. In particular, in fermented foods, NSLAB can accumulate BAs and strains of lactobacilli, enterococci, lactococci, pediococci, streptococci, and leuconostocs have been associated with high levels of these compounds [118]. Genetic studies have revealed that many of these strains harbor genes or operons coding for decarboxylating enzymes or other pathways implicated in BA biosynthesis [9,64].

Hereafter, the main LAB genera associated with fermented products and involved in BA production in vitro or in situ are described.

### 3.1. Enterococcus

The *Enterococcus* genus has not been yet classified as safe for human consumption since it neither is recommended for the Qualified Presumption of Safety (QPS) list nor have GRAS status. Most of the species harbor a series of virulence factors and antibiotic resistance and they have been associated with several infections, having the ability to mediate gene transfer with different genetic elements, including plasmids, phages and conjugative transposons [119,120]. The role of enterococci in fermented foods remains controversial. They show remarkable ecological adaptability and ability to grow in adverse conditions. Due to their tolerance to salt and low pH, they are highly adapted to several food systems and they are also involved in the fermentation process of traditional cheeses and dry sausages [121]. Moreover, some *Enterococcus* strains show probiotic features [122] or can improve sensorial properties of dairy products when added as adjunct starters, taking part to flavour generation through proteolytic and lipolytic activities and the accumulation of C4 metabolites such as diacetyl, acetoin or 2, 3-butanediol [123,124]. In addition, their ability to biosynthesize bacteriocins with a wide-range effectiveness on pathogenic and spoilage bacteria is known [125].

Nevertheless, enterococci presence in fermented foods has been associated with the production of BAs (mainly tyramine) and this activity has been reported for strains belonging to different species isolated from meat, cheese, fish, wine and human faeces [54,126,127,128,129,130,131,132]. However, not all the strains able to decarboxylate tyrosine were characterized by the same phenotypic potential in relation to the kinetics of tyramine accumulation [15] (Table 1).

Enterococci have been recognized as important part of the natural microbiota in many artisanal cheeses and, in some cases, they can predominate over lactobacilli and lactococci [133]. Usually, enterococci are not present in starter cultures and thus all species of this genus isolated from cheese samples represent contaminating microbial communities, and can include aminobiogenic strains. The most common species found in milk are *Enterococcus faecium*, *Enterococcus durans* and *E. faecalis* but, even if with minor extent, *Enterococcus casseliflavus* may also be isolated [123] and mostly of the strains belonging to these species and isolated from cheese have been identified as tyramine producers [132]. Several authors found a relation between the enterococci counts and the concentrations of tyramine [134,135,136,137] and putrescine [67] in dairy products.

Burdychova and Komprda [138] detected tyraminogenic isolates from cheese belonging to *E. durans*, *E. faecium*, *E. faecalis* and *E. casseliflavus* species. Rea et al. [139] studied the effect of six strains of *E. faecalis*, *E. faecium*, *E. durans* and *E. casseliflavus* species on tyramine production in Cheddar cheese during manufacturing and ripening and found that all strains, except *E. casseliflavus*, produced this BA, with *E. durans* responsible for the highest concentration after 9 months of ripening at 8 °C. Enterococcal strains isolated from an Italian cheese and from raw goat milk showed high decarboxylase activity with tyrosine and phenylalanine as substrates [59,136]. Kalhotka et al. [140] investigated the decarboxylase activity of enterococci isolated from goat milk and found that all the tested strains, identified as *Enterococcus mundtii*, *E. faecium* and *E. durans*, showed significant tyrosine and arginine decarboxylase activity, in relation to temperature and time of incubation. Martino et al. [141] studied safety features of four enterococcal strains isolated from a regional Argentinean cheese founding that these strains possessed *tdc* gene cluster, even if only two of four strains gave a positive result in Bover-Cid and Holzapfel decarboxylase screening medium [142]. These authors hypothesized the possibility that this pathway was not active, although all the strains possessed the complete decarboxylase cluster.

The presence of enterococci able to produce BAs is a relevant food issue also in meat products, despite their recognized role in the development of sensory properties of fermented products particularly in sausage [143,144]. In fact, enterococci are constituents of the natural microbiota of raw meat and of many fermented meat products [145], with *E. faecium* and *E. faecalis* being the predominant species, followed by *Enterococcus hirae*, *E. durans* and *E. mundtii* [122]. For this reason, dry fermented sausages can easily accumulate high levels of BAs, especially tyramine, putrescine and cadaverine [82]. In contrast, histamine is usually scarcely found in fermented sausages [146].

Landeta et al. [147] found that 79% of *E. faecium* strains isolated from Spanish dry-cured sausages were able to produce tyramine and that some strains were PCR-positive for the presence of the tyrosine decarboxylase gene, but were not able to accumulate this BA, due to the absence of gene expression. These results were in agreement with those obtained by Komprda et al. [37] who reported that 88% of enterococcal strains isolated in ripened fermented sausages and belonging to *E. faecium* and *E. faecalis* species, possessed *tdc* sequences. These authors found also that 71% of enterococcal isolates had *hdc* gene sequence, assuming that the decarboxylation pathway (producing proton motive force) gives the strains a competitive advantage in nutrient-depleted conditions and acidic environments, such as fermented sausages at the end of ripening. The potential of different indigenous enterococci to contribute to BA formation in spontaneously fermented game meat sausages has been reported also by Maksimovic et al. [148], who found that 100% of *E. durans* and about 7% of *E. casseliflavus* possessed *tdc* genes. Iacumin et al. [62] indicated enterococci able to accumulate large amount of BAs as responsible for spoilage in goose sausages produced in the north of Italy. In fact, despite the addition of starter, enterococci grew during ripening and produced a large amount of BAs. This ability was confirmed in vitro, since all the isolates (*n* = 100), belonging to the species *E. faecium* and *E. faecalis*, were able to decarboxylate amino acids and produce BAs. In particular, all the strains produced histamine, and 60 out of 70 *E. faecium* and 25 out 30 *E. faecalis* strains produced cadaverine and 10 isolates belonging to both species produced tyramine.

Enterococci has been reported as mainly responsible for tyramine accumulation in wine during malolactic fermentation, together with some *Lactobacillus* species [18,127,149,150]. These latter authors isolated *E. faecium* strains during malolactic fermentation of red wine and demonstrated that, although all the isolates harbored decarboxylase genes, only five strains were able to survive under the harsh conditions found in wine (high ethanol content and low pH), leading to a higher concentration of BAs in samples, including tyramine, histamine and 2-phenylethylamine.

*E. faecium* and *E. faecalis* have been considered responsible also for BA production in fermented soybean food [151] and in tofu [152].

Although aminobiogenic capability is reported to be strain dependent, Ladero et al. [54] suggested that tyramine and putrescine biosynthesis is a species level trait in *E. faecalis*. In fact, independently of the origin, several strains have been identified as BA producers. Moreover, PCR results demonstrated that the same genetic organization was present in all the tested strains and their decarboxylase clusters were independently located in the chromosome, with flanking regions showing within-species homogeneity.

In *E. faecalis*, putrescine is formed from agmatine by the AgDI pathway, which is repressed by carbon source, suggesting a role in the energy production [153]. Perez et al. [154] studied the possible co-regulation among TDC and AgDI pathways in *E. faecalis*. They investigated firstly the tyrosine effect on the *tdc* cluster transcription of *E. faecalis* by microarray experiment, highlighting, in the presence of tyrosine, an over-expression of *tdcA*, *tdcP*, and *nhac-2* genes and a repression of *tyrS*. Bargossi et al. [15,155] have also demonstrated the same effect in other *E. faecalis* strains. Moreover, Perez et al. [154] showed that tyrosine induced putrescine biosynthesis genes, as confirmed by reverse transcription quantitative PCR (RT-qPCR) results. On the other hand, this effect was not observed in the mutant strain, which was unable to decarboxylate tyrosine and produce tyramine, showing that *tdc* cluster was involved in the tyrosine induction of putrescine biosynthesis.

Recently, some authors demonstrated that also *E. mundtii* possesses the capability to produce both tyramine and 2-phenilethylammine [107]. The genetic organization indicated that the tyramine-forming pathway in *E. mundtii* is similar to that found in phylogenetically closer enterococcal species, such as *E. faecium*, *E. hirae* and *E. durans*. The gene Na^+^/H^+^ antiporter (*nhaC*) that usually follows *tyrP* was missing. However, the analysis of the available data on *E. mundtii* genome revealed the presence of a further region that includes two genes encoding for an additional pyridoxal phosphate (PLP)-dependent decarboxylase and an amino acid permease, correlated with the tyrosine decarboxylating potential of this species.

In any case, tyramine is often accumulated by enterococci in high amounts already during the late exponential growth, before stationary phase, suggesting that this decarboxylation activity is not necessarily a response to starvation or nutrient depletion, and no competition between sugar catabolism and amino acid decarboxylation was observed [15,17]. In particular, these latter authors tested the ability to accumulate tyramine and 2-phenylethylamine by two strains of *E. faecalis* and two strains *E. faecium* in two culture media added or not with tyrosine. They demonstrated that, although all the tested enterococcal strains possessed a TDC pathway, they differed in BA accumulation level and in the expression rate of *tdc* gene, underlining the extremely variable decarboxylating potential of strains belonging to the same species, suggesting strain-dependent implications in food safety.

Environmental factors such as pH, temperature and NaCl concentrations can affect BA production in enterococci and several studies on decarboxylase activity of *Enterococcus* spp. in different conditions have been carried out. Gardini et al. [156] investigated the combined effects of temperature, pH and NaCl concentration on tyramine production by the strain *E. faecalis* EF37, finding that production of tyramine was mainly dependent on cell number. Moreover, these authors reported that this strain was able to accumulate also 2-phenylethylamine. A study regarding EF37 *tyrDC* expression revealed that stress could induce greater tyrosine decarboxylase activity, suggesting that suboptimal environmental conditions could lead to a higher tyrosine production, not necessarily associated with cell growth. This could be explained with the physiological role of this biochemical pathways associated with the survival of LAB in hostile environments [157]. Acidic conditions favored tyramine production in an *E. durans* BA-producing strain isolated from cheese [158] and in *E. faecium* [16,26], demonstrating the role of tyrosine decarboxylation in pH homeostasis. On the other hand, transcriptional studies of the *tdc* cluster in *E. durans* 655 showed a pH regulation of tyramine biosynthesis, being the gene expression quantification during the exponential phase induced by high concentrations of tyrosine, under acidic conditions [159].

Bargossi et al. [105] investigated the diversity of tyramine production capability of two *E. faecalis* and two *E. faecium* strains in buffered systems in relation to their genetic characteristics and to pH, NaCl concentration and incubation temperature, comparing the results with those obtained with a purified tyrosine decarboxylase under the same conditions. They found that TDC activity was greatly heterogeneous within the enterococci, being *E. faecalis* EF37 the most efficient in tyramine accumulation. This heterogeneity depended on different genetic determinants, regulation mechanisms and environmental factors, above all incubation temperature.

A reduced transcription of genes involved in tyramine production was observed in the presence of 6.5% of NaCl in *E. faecalis* [160]. Also Bargossi et al. [105] showed that *E. faecalis* partially reduced its tyraminogenic potential passing from 0 to 5% of NaCl but the decarboxylation activity did not change significantly increasing NaCl concentration up to 15%. On the contrary, Liu et al. [161] demonstrated that NaCl stress can upregulate the expression of *tyrDC* and *tyrP* to improve the tyramine production of a single *E. faecalis* strain under certain conditions.

### 3.2. Lactobacillus

Lactobacilli are reported to be strong BA producers in different fermented foods [94].

In fermented sausages, beside to enterococci, the main tyramine producers among LAB are strains belonging to *Lactobacillus curvatus* species, which is, together with *Lactobacillus sakei*, the predominant *Lactobacillus* species in fermented meat products [162,163]. In fact, the majority of *L. curvatus* strains isolated from meat were reported to be tyramine producers [126]. However, Bover-Cid et al. [126] reported also some strains of *Lactobacillus paracasei*, *L. brevis*, and *L. sakei* isolated from pork-fermented meat as tyramine forming. Pereira et al. [164] demonstrated tyrosine and ornithine decarboxylase activities in *Lactobacillus homohiochii* and *L. curvatus* isolated from a Portuguese traditional dry fermented sausage.

Freiding et al. [165] screened *L. curvatus* strains from different origins, finding strain dependent tyrosine decarboxylase activity. Moreover, although *L. sakei* is usually described as non- aminogenic, histidine decarboxylase activity in one *L. sakei* strain has been evidenced [81,126,166].

*Lactobacillus parabuchneri* and *L. buchneri*, present as contaminants in fermented meat products, can produce histamine [167].

LAB populations were isolated from dry fermented sausages produced with different starters and using two spice mixtures in different process time by Kompdra et al. [37]. Tyrosine-decarboxylase and histidine-decarboxylase DNA sequence was identified in 44% and of 16% of lactobacilli isolates, respectively. In particular, several *Lactobacillus plantarum*, *L. brevis* and *Lactobacillus casei/paracasei* strains were identified as tyramine and histamine producers in the sausages analysed.

Although several microorganisms in cheese, including Gram negative bacteria, are able to produce BAs, *Lactobacillus* species, such as *Lactobacillus helveticus*, *L. buchneri* and *L. curvatus*, can be responsible for their accumulation in such products [38,138,168]. For example, specific strains of *L. buchneri* and *L. parabuchneri* harbor the histidine decarboxylase enzyme and can develop high levels of histamine, even at refrigerate temperature [39,169]. Wüthrich et al. [167] analysed several *L. parabuchneri* strains isolated from cheeses, finding some histamine positive among them. Moreover, these authors determined the complete genome of a histamine positive strain, showing that *hdc* gene cluster is located in a genomic island, transferred within the *L. parabuchneri* species. Diaz et al. [40] isolated, for the first time, 25 histamine-producing *Lactobacillus vaginalis* strains and sequenced *hdc* gene cluster and its flanking regions for a representative strain (*L. vaginalis* IPLA11050). These authors suggested that *hdc* locus was localized in the chromosome and, being the flanking regions the same in all histamine-producing *L. vaginalis* tested strains, histamine production has been suggested to be a species level trait. In addition, the organization of the examined genes was the same described for *Lactobacillus reuteri* [41], *L. buchneri* [111] and *L. hilgardii* [33] but differed to that of *S. thermophilus*.

*L. brevis* tyramine-producing strains have been isolated from cheeses by several authors [170,171] and this feature has been described as a strain-level trait (perhaps horizontally acquired) in *L. brevis*. Pachlová et al. [172] assessed the development of BA content in model cheese samples individually inoculated with two BA producing NSLAB strains of *L. curvatus* subsp. *curvatus* and *L. paracasei*, demonstrating the ability of these strains to accumulate tyramine up to 200 mg/kg in real dairy products during a 90 days ripening period. Yilmaz and Görkmen [173] demonstrated the capability to produce tyramine by a *L. plantarum* strain in yogurt and highlighted a possible indirect effect of *Lactobacillus delbrueckii* subsp. *bulgaricus* on accumulation of tyramine in the yoghurts, due to its synergistic interactions with tyraminogenic LAB strains.

It has been reported that non-starter *L. brevis* and *L. curvatus* are able to produce both tyramine and putrescine [174]. Although the ODC pathway has been described in several LAB, including strains of *L. brevis* [20], this pathway is not commonly used by dairy bacteria [64,67,175]. Ladero et al. [174] confirmed this aspect, showing that the detected putrescine-producing lactobacilli used AgDI pathway. Lucas et al. [101] found that *L. brevis* IOEB 9809 produced putrescine from agmatine but not from arginine, indicating the lack of a pathway converting arginine into agmatine. Moreover, it has been suggested that in *L. brevis* the AgDI genetic determinants are linked to those of the TDC pathway and are located in an acid resistance mechanism locus, probably acquired by horizontal gene transfer [20].

Several native lactobacilli, together with *O. oeni* and *Pediococcus parvulus* strains, are responsible for BA accumulation in wine. Their formation in wine depends on several conditions such as precursor amounts and the presence of specific decarboxylase-positive species and strains [176,177] and they are produced mainly during malolactic fermentation, particularly due to the presence of *L. brevis* and *L. hilgardii* [178,179,180]. Landete et al. [42] reported aminobiogenic potential of LAB isolated from wine samples, evidencing *L. mali* strains able to produce histamine, *L. brevis* strains able to accumulate tyramine and 2-phenylethylamine and a *L. hilgardii* strain showing histamine, tyramine, 2-phenylethylamine and putrescine production ability. On the other hand, the HDCs of *L. hilgardii* isolated from wine are well documented [33]. The enhancing effects of lower pH on histamine production (as responses to acidic stress) was observed in *L. brevis* [181]. Henríquez-Aedo et al. [182] reported that *Lactobacillus rhamnosus* was unexpectedly the predominant species in the vinification process of Chilean Cabernet Sauvignon wines and that it was mainly responsible for histamine accumulation in the products, presenting a significantly higher BA formation capability with respect to *O. oeni* isolated from the same samples. Arena and Manca de Nadra [21] studied a *L. plantarum* strain able to produce putrescine from arginine and ornithine while Moreno-Arribas et al. [183] found two wine strains of *L. buchneri* able to form putrescine via ornithine decarboxylase.

In wines, tyrosine decarboxylase has been associated with *Lactobacillus* spp., particularly *L. brevis* strains [84]. The same authors purified this pyridoxal 5P-phosphate dependent enzyme and showed it was highly substrate-specific for l-tyrosine and had an optimum pH of 5 [184]. The ability of a *L. plantarum* strain isolated from a red wine to produce tyramine from peptides containing tyrosine, especially during the late exponential growth phase, has been demonstrated [55] and *tdc* genes shared 98% identity with those in *L. brevis* consistent with horizontal gene transfer from *L. brevis* to *L. plantarum*. Arena et al. [185] assessed the expression of *L. brevis* IOEB 9809 *tdc* and *aguA1* genes during wine fermentation and evaluated the effect of substrate availability and pH on it, as well as on BA production, showing that the strain was able to produce both tyramine and putrescine. In addition, qRT-PCR analysis suggested a strong influence of substrate availability on the expression of BA pathway genes while less evident was pH influence. Afterwards, Lucas and Lonvaud-Funel [186] and Lucas et al. [53] reported for the same strain the complete *tdc* sequences, describing four complete genes (*tyrS*, *tyrDC*, *tyrP* and *nhaC*).

The BA production ability in different *Lactobacillus* strains, isolated from wine and cider, and their metabolic pathway were explored by Constantini et al. [68]. Their results demonstrated that most of the *L. brevis* analyzed harbor both *AgDI* and *tdc* genes and were tyramine and putrescine producers. Interestingly, these authors detected *hdc* genes in a *L. casei* strain isolated from cider.

Beer spoilage LAB showed several metabolic strategies to grow in nutrient poor environment, with acidic pH and hop presence among which BA production, contributing to energy supply and pH homeostasis, has been highlighted [187]. In particular, heterofermentative *L. brevis* strains accumulated tyramine and ornithine while *Lactobacillus lindneri* and *Lactobacillus paracollinoides* beer spoiling agent displayed ornithine and histamine production, respectively. Strains belonging to this latter species have been indicated as new potential histamine- and putrescine-producers in cider analysed by Ladero et al. [188]. Also Lorencová et al. [63] demonstrated that *L. brevis* strains isolated from beer can be a tyramine source in these products. The same authors showed the possibility to produce BAs by some probiotic strains belonging to *L. rhamnosus* species, opening a serious concern about the need to investigate the decarboxylation activity of probiotic or functional cultures before their use.

Recently, the capability to produce putrescine of a *Lactobacillus rossiae* strain, previously isolated from sourdough, has been reported [69]. This species is widely distributed in this fermented food [189] but the possibility of *L. rossiae* sourdough strains to produce BAs was not previously shown. In fact, only a strain of this species isolated from a wine starter has been described as histamine producer [190]. del Rio et al. [69] showed that *L. rossiae* strain accumulated this BA via the ODC pathway and the genetic organization and transcriptional analysis of the gene cluster identified the *odc* and *potE* genes forming an operon that is transcriptionally regulated by ornithine in a dose-dependent manner. Moreover, putrescine production via the ODC system improved the survival of *L. rossiae* by counteracting the cytoplasm acidification when the cells were subjected to acidic conditions, providing a biochemical defense mechanism against acidic environments. For this reason, this strain could easily produce putrescine during the fermentation process and the potential presence in sourdough of other BA-producing microorganisms cannot be ruled out.

The first description of ODC system in LAB was reported for *L. saerimneri* 30a [191]. Further investigation on this strain evidenced the presence of a unique genomic organization in which *odc* does not have an adjacent specific transporter gene but a three-component decarboxylase system with a lysine decarboxylase gene (*aadc*) and a promiscuous amino acid-amine transporter gene (*aat*), appearing atypical from those of other LAB [192].

### 3.3. Streptococcus

Although certain *Streptococcus* species are responsible for many disease (i.e., meningitis, bacterial pneumonia, endocarditis, necrotizing fasciitis etc.) many streptococcal species form part of the human microbiota and are important for fermented foods. In fact, *S. thermophilus* is often employed as selected starter culture and it is important for the dairy industry, since it is one of the principal components of many natural cultures used in fermented products such as hard cooked or pasta filata cheeses, yogurt and Cheddar [193]. This species is usually present in high numbers in the first steps of cheese-making and its relationships with BAs, which mainly accumulate during ripening, has been longer neglected. In fact, despite its wide industrial use, there are few papers regarding the decarboxylating potential of this species. Nevertheless, some BA producing strains have been identified and studied in recent years and several screenings on aminobiogenic potential of *Streptococcus* spp. strains have been performed. Ladero et al. [174] studied the BA producing ability of 137 strains of starter and NSLAB belonging to nine species of the genera *Lactobacillus*, *Lactococcus*, *Streptococcus* and *Leuconostoc* (all isolated from artisanal cheeses) in liquid media supplemented with the appropriate precursor amino acid by Ultra-High Performance Liquid Chromatography technique. Moreover, assessing the presence of key genes involved in the biosynthetic pathways of the target BA, they found that two *S. thermophilus* strains possessed *hdc* genes, although they were unable to synthesize histamine in broth. Also strains belonging to *Streptococcus macedonicus* species, isolated from Greek Kasseri cheese, showed tyramine production [194]. Some authors demonstrated that *Streptococcus mutans* expressed an agmatine deiminase system, encoded by the agmatine-inducible *aguBDAC* operon, which was induced in the presence of agmatine and was regulated by carbon catabolite repression. This metabolism was proposed to augment the acid resistance properties and pathogenic potential of *S. mutans*, etiological agent of dental caries and acid tolerance in oral biofilms [195,196].

Elsanhoty and Ramadan [197] reported the presence of *tdc*, *hdc* and *AgDI* genes in a *S. thermophilus* strain. Buňková et al. [48] studied BA production capability of selected technological important LAB belonging to *Lactococcus*, *Lactobacillus* and *Streptococcus* genera. Among these strains, one *S. thermophilus* of 11 was able to produce tyramine. Gezginc et al. [198] investigated the BA production capability of *S. thermophilus* isolates in homemade natural yogurt, evidencing the presence of *hdcA* gene in several strains, although it was poorly correlated with histamine production in the decarboxylase medium. Yilmaz and Gökmen [173] investigated tyramine formation during yoghurt fermentation, focusing on interaction between a *S. thermophilus* strain and some *Lactobacillus* species The streptococci cells were able to produce tyramine depending on the fermentation conditions and synergistic interactions between *S. thermophilus* and *L. delbrueckii* subsp. *bulgaricus* were found in terms of BA accumulation.

The decarboxylating potential of the strain *S. thermophilus* NCFB2392 in lysine decarboxylase broth has been reported [199]. This strain was able to accumulate mostly putrescine, cadaverine and agmatine, and, when co-cultered with other BA producer Gram negative strains, had synergistic or antagonistic effect on BA concentrations. In fact, it caused 2-fold lower cadaverine production by *Salmonella* Paratyphi A and stimulated tyramine accumulation of *Escherichia coli*.

A high number of *S. thermophilus* strains have been investigated for tyramine production by La Gioia et al. [49]. Only the strain 1TT45, isolated from Taleggio cheese, demonstrated the capability to accumulate tyramine in broth. For this strain, a tyrosine decarboxylase (*tdc*A) gene was identified, with a nearly identical sequence to a *tdc*A of *L. curvatus*, indicated a horizontal gene transfer event. In the same work *tdcA* expression level and the production of tyramine were evaluated under different conditions during 7 days of incubation in skim milk. High transcript levels were evidenced only at the seventh day in presence of tyrosine, showing that the ability of *S. thermophilus* 1TT45 to form this BA depends on precursor availability in the culture medium, due to the incapability of this species to release peptides and free amino acids from milk proteins when grown in pure culture. On the other hand, the presence in cheeses of highly proteolytic LAB species would likely allow tyramine formation by *S. thermophilus*.

Calles-Enríquez et al. [35] observed two *S. thermophilus* strains able to produce histamine and reported their complete *hdc* gene cluster organization. This cluster began with the *hdcA* gene, was followed by a transporter (*hdcP*) and ended with the *hdcB* gene, located in the chromosome and orientated in the same direction.

The gene order of *hdcAPB* operon is similar to *Staphylococcus capitis* and *Clostridium perfringens*, which, however, lacks *hdcB* [200]. Transcriptional analysis of the *hdc* cluster revealed the maximum expression during the stationary growth phase, with high expression levels correlated with high histamine concentration. In the same work, also some factors affecting histamine biosynthesis and histidine-decarboxylating gene (*hdcA*) expression were studied. In particular, low temperature incubation determined lower levels of histamine in milk than in samples kept at 42 °C. This reduction was attributed to a reduction in the activity of the HDC enzyme itself rather than a reduction in gene expression or the presence of a lower cell number.

The occurrence of a histidine decarboxylase gene (*hdc*A) was demonstrated also in five among 83-screened *S. thermophilus* strains by Rossi et al. [85]. The sequence of the *hdc*A gene and closest flanking regions were determined for the strain PRI60, which produced the highest amounts of histamine. This strain synthesized HDC enzyme in milk even in the absence of histidine and it remained active also in cell-free extracts. Tabanelli et al. [201] continued the study of the histamine potential of PRI60 strain, testing histamine accumulation by cells or crude enzyme preparations with respect to factors related to dairy products, reporting a histamine concentration increase concomitantly with the cell growth. Moreover, HDC was mostly active at pH 4.5 and salt concentration up to 5% (*w*/*v*) did not affected enzyme activity. These authors evidenced enzyme thermal resistance up to temperature resembling low pasteurization, showing the risk of the presence of histaminogenic *S. thermophilus* strains in products from raw or mildly heat-treated milk. In fact, this strain was able to accumulate histamine in experimental cheeses, both when inoculated as starter or as cell-free crude enzyme preparations, highlighting that histamine formation by *S. thermophilus* in artisanal cheeses must not be overlooked, especially during typical production practices [31].

### 3.4. Lactococcus

Lactococci are among the most important LAB involved in the dairy industry and some species, including *Lc. lactis* subsp. *lactis*, *Lc. lactis* subsp. *lactis* biovar *diacetylactis* and *Lc. lactis* subsp. *cremoris*, play a critical role in the manufacture of many fermented dairy products [202]. As well as starters, they have been proposed as bioprotective cultures in the food industry and, for these reasons, safety criteria (such as BA production) should be evaluated.

Despite their QPS status (recognized by EFSA) and their generally regarded as safe (GRAS) status (recognized by FDA), some *Lc. lactis* have been reported to have aminobiogenic activity, both in vitro and in real systems. In fact, several strains of *Lc. lactis* subsp. *lactis* and *Lc. lactis* subsp. *cremoris* able to produce putrescine from agmatine via the AgDI pathway have been identified and these species are known to be, together with *E. faecalis*, *E. hirae*, *L. brevis* and *L. curvatus*, the main putrescine producers in dairy products [65,67,174]. The analysis of the *AgDI* cluster and their flanking regions revealed that the capability to produce putrescine via the AgDI pathway could be a specific characteristic that was lost during the adaptation to the milk environment by a process of reductive genome evolution [65]. The AgDI pathway increases the growth of *Lc. lactis* and causes the alkalinization of the culture medium, although it does not seem to be an acid stress resistance mechanism [30]. Linares et al. [203] investigated the role of *aguR* gene in putrescine formation in *Lc. lactis* subsp. *cremoris* CECT 8666, founding that it is essential for putrescine biosynthesis and it is transcribed independently of the polycistronic mRNA encoding the catabolic genes. Moreover, the transmembrane protein encoded by *aguR* can act as a transcription activator of the putrescine biosynthesis operon in response to the agmatine concentration. The same strain was tested in experimental Cabrales-like cheeses with different NaCl concentrations [204]. These authors evidenced that reducing the NaCl concentration of cheese led to increased putrescine accumulation and that NaCl was able to reduce the transcription of the *aguBDAC* operon, even if no effect on the transcription of *aguR* was recorded. The same authors investigated the effect of extracellular pH on putrescine biosynthesis and on the genetic regulation of the AgDI pathway of CECT 8666 strain. They showed increased putrescine biosynthesis at pH 5, when the transcription of the catabolic operon via the activation of the *aguBDAC* promoter P*aguB* was induced and a protection against acidic external conditions was reached through the counteraction of cytoplasm acidification [205].

It has been reported that *Lc. lactis* can produce other BAs in addition to putrescine. Martins Perin et al. [59] evaluated the BA production of bacteriocinogenic lactococci strains isolated from raw goat’s milk reporting tyramine and 2-phenylethylamine accumulation capability in some of them. The decarboxylase activity of two aminobiogenic strains of *Lc. lactis* subsp. *cremoris* employed as starters in a model system of Dutch-type cheese was studied during a 90 day ripening period [206]. While in the control samples the amount of BAs was negligible, lactococci accumulated about 500 and 800 mg/kg of tyramine and putrescine, respectively. The putrescine decarboxylase activity observed in the model samples of cheeses with the inoculated strains was consistent with the results by Santos et al. [207].

### 3.5. Oenococcus and Pediococcus

*Pediococcus* spp. are often isolated from a large variety of plant materials and are involved in spontaneous fermentation of silage, sauerkraut, beans, cucumbers, olives, and cereals [208]. In addition, pediococci are also associated with fermented sausages and cheese, where selected strains of *Pediococcus pentosaceus* and *Pediococcus acidilactici* are often exploited as commercial starters, to control the development of undesired and pathogenic microbiota given their bacteriocinogenic features [209]. However, they can act also as spoilers in several fermented foods such as beer, wine and cider, causing turbidity, acidic off-tastes, adverse flavors and accumulating undesirable compounds [210].

*O. oeni* is a wine-associated LAB, considered the dominant species during the malolactic fermentation and possesses remarkable adaptability to harsh physico-chemical conditions. Several *O. oeni* strains have been described as BA producers and therefore many authors considered this species, together with other LAB species such as *L. hilgardii* and *Pediococcus*, as responsible for histamine presence in wine [33,36,43,108,176].

Lonvaud-Funel and Joyeux [44] isolated for the first time a strain of *O. oeni* able to produce histamine via histidine decarboxylase from a wine from the Bordeaux area. Subsequently, Coton et al. [108] purified and characterized this enzyme, concluding that it requires pyridoxal-5-phosphate as cofactor. Other authors have also shown that histamine-producing strains of *O. oeni* are frequent in wine [211] but this feature was strain dependent and in some strains no BA potential has been found [176]. For this reason, the role of *O. oeni* in BA accumulation in wine is still controversial [212]. As a possible explanation for these discrepancies, it has been suggested that the *hdc* genes are located on a large and possibly unstable plasmid and that culture collections will lose the capability to produce histamine in laboratory subcultures because of the loss of this unstable plasmid [36]. In the LAB isolated from wine by Landete et al. [42] mostly of *O. oeni* and *P. parvulus* harbored *hdc* genes and pediococci produced high level of histamine in synthetic medium, showing the highest histaminogenic potential within the tested genus. Nevertheless, in the same conditions *O. oeni* showed lower levels of histamine production, that were even lower when these strains were tested in wine samples. Berbegal et al. [213] showed the presence of both histamine producer and non-producer strains in a Spanish red Ribera del Duero wine and proposed a non histaminogenic strain to be used as starter reducing of 5-fold histamine content in inoculated wine than the non-inoculated control. It has been further demonstrated that, after one year, the barrel-ageing histamine concentrations were 3-fold lower in the inoculated vat than in the non-inoculated one.

Landete et al. [214] studied the influence of enological factors on the *hdc* expression and on HDC activity in *P. parvulus* and *O. oeni*. Gene expression was lowered by glucose, fructose, malic acid, and citric acid, whereas ethanol enhanced the HDC enzyme activity, so that the conditions normally occurring during malolactic fermentation and later on, could favor histamine production. On the other hand, Gardini et al. [177] evaluated the interactive effect of some variables on the BA production of *O. oeni*, demonstrating that high ethanol amounts and low concentration of pyridoxal-5-phosphate reduced their accumulation while higher pH enhanced BA concentrations. In addition, the SO_2_ effect on tyramine accumulation depended also on other variables.

In cider, where a microbiological stabilization after malolactic fermentation is not performed, indigenous heterofermentative LAB constitute the predominant microbiota and several species such as oenococci and pediococci (beside to lactobacilli) are able to produce BAs [18,215]. Some pediococci strains isolated from wine and ciders showed the presence of decarboxylase genes, i.e., *AgDI* cluster in *P. parvulus* and *P. pentosaceus* [18,68,188]. In this latter work, also *O. oeni* strains had *AgDI* genes and the authors found a few discrepancies between phenotypic and genotypic data. On the other hand, the identification of an *odc* gene in a putrescine producer *O. oeni* strain has been reported by Marcobal et al. [70]. Later, the *odc* gene was also identified and sequenced in three *O. oeni* wine strains and in two *O. oeni* cider strains [216] and the sequencing of the complete *odc* gene from *O. oeni* and *L. brevis* showed an 83% identity [19].

Low production of cadaverine and tyramine was also found in a *Pediococcus* spp. strain isolated from beer [63] and Izquierdo-Pulido et al. [217] reported *Pediococcus* genus to be mainly responsible for tyramine accumulation in beer.

### 3.6. Other Genera: Weissella, Carnobacterium, Tetragenococcus, Leuconostoc, Sporolactobacillus

Leuconostocs are LAB associated with plants and decaying plant material, often detected in various fermented vegetable products but also in foods of animal origin [218]. Some species such as *Leuconostoc carnosum*, *Leuconostoc gasicomitatum*, and *Leuconostoc gelidum* have often been associated with food spoilage and some strains have been found to be decarboxylase positive. Although it is known that AgDI pathway has been demonstrated in *Leuconostoc mesenteroides* [18], some strains isolated from wine have been suggested to produce putrescine exclusively from arginine via the arginine deiminase pathway (ADI) pathway, given to the selective effect of the ecological niche on BA biosynthesis pathway [115,219]. Recently, based on current knowledge and QPS/GRAS/dairy (IDF) safety criteria guidelines, the safety of different LAB candidate antifungal bioprotective strains has been evaluated finding a tyramine-producer *Leuc. mesenteroides*. This result confirmed the importance to test decarboxylase activity before considering a candidate strain for use as a bioprotective agent in food products [220]. Dairy strains of leuconostocs have been associated with high levels of BAs in cheese and other dairy products and tyramine and 2-phenylethylamine production in *Leuconostoc* strains isolated from dairy products have been reported [32,221]. Moreno-Arribas et al. [183] found that *Leuc. mesenteroides* may also be responsible for tyramine production in wines and Landete et al. [42] isolated a wine strain belonging to this species able to produce histamine.

This genus can be also implicated in beer BA accumulation. In fact, some authors studied the occurrence of aminobiogenic strains during a craft brewing process, highlighting the presence of *Leuc. mesenteroides* possessing *tdc*, *hdc*, *odc* decarboxylase genes and able to produce tyramine in wort and beer [61]. *Leuc. mesenteroides* ssp. *mesenteroides* isolated from meat, fermented sausages and cheeses was able to form putrescine and cadaverine [56].

*Weissella* are heterofermentative LAB which occur in a wide range of habitats, i.e., milk, plants and as well as from a variety of fermented foods such as European sourdoughs and Asian and African traditional fermented vegetables. They can be involved in such traditional fermentations and some strains of *Weissella confusa* and *Weissalla cibaria* can produce copious amounts of dextran but strains of certain *Weissella* species are known as opportunistic pathogens involved in human infections [222]. Moreover, some *Weissella* strains have been demonstrated to be able to produce BAs in fermented foods. *Weissella viridescens* isolated from Tofu-misozuke, a traditional Japanese fermented food, resulted tyramine producers and several strains isolated from kimchi belonging to *W. cibaria*, *W. confusa* and *Weissella paramesenteroides* produced multiple BAs, including tyramine and histamine [45,152]. On the other hand, Pereira et al. [71] demonstrated that a *Weissella halotolerans* strain combines an ornithine decarboxylation pathway and an arginine deiminase pathway, leading to the accumulation of putrescine and producing a proton motive force.

*Carnobacterium* spp. can be found in vacuum or modified atmosphere packed, refrigerated raw or processed meat products and lightly preserved fish products, milk, and certain types of soft cheese [223]. Although *Carnobacterium divergens* and *Carnobacterium maltaromaticum* have been demonstrated to be bacteriocin producers, able to inhibit *Listeria monocytogenes*, some strains appear to display undesirable properties such as amino acid decarboxylation activities. In fact, these two species can produce tyramine while strains belonging to *C. divergens*, *Carnobacterium gallinarum*, *Carnobacterium maltaromaticum* and *C. mobile* can possess ADI pathway [57,104,223]. Curiel et al. [224] studied the BA production capability by LAB and enterobacteria isolated from fresh pork sausages and reported that all the tyramine-producer isolated strains were molecularly identified as *C. divergens*, whose abundance depended from the different packaging conditions. All these strains presented the *tdc* genes. Coton et al. [51] identified the gene encoding a putative tyrosine decarboxylase in *C. divergens*, evidencing the presence of three putative open reading frames (*tyrS*, *tyrDC* and amino acid transporter *PotE*) which showed the strongest homologies with *E. faecium* (94% identity, 98% homology) and *E. faecalis* (85% identity, 92% similarity) and exhibited conserved domains characteristic of the group II (PLP-dependent) decarboxylase family.

Other genera less frequent in fermented foods may also be involved in BA production.

*Tetragenococcus* is a halophilic facultative aerobic homofermentative coccus, which cannot be readily distinguished from members of the genus *Pediococcus* and which can play a role in halophilic fermentation processes such as the production of soy products, brined anchovies, fish sauce and fermented mustard or can constitute the dominant microbiota in concentrated sugar-concentrated juice [225]. Recently, the safety of 49 *Tetragenococcus halophilus* strains isolated from the Korean sauce doenjang has been assessed. The isolates produced higher tyramine level than reference strains and similar cadaverine, histamine, and putrescine production patterns [72]. A *T. muriaticus* strain isolated from fish sauce produced histamine during the late exponential growth phase, reaching a maximum production of this BA at 5–7% of NaCl, and was able to maintain a histidine decarboxylase activity also in the presence of 20% of salt [46].

Recently, a sporulating LAB, belonging to a novel species of *Sporolactobacillus* genus and isolated from cider must, harbored *tdc* gene, which showed the same organization as already described genes found in other tyramine-producing LAB. Moreover, genes showing the highest identities with mobile elements surrounded the *tdc* operon, suggesting that the tyramine-forming trait was acquired through horizontal gene transfer [58].

## 4. Conclusions

Biogenic amines can accumulate in high concentrations in fermented foods due to microbial activity and can cause toxic effects in consumers. LAB are considered mainly responsible for BA accumulation in these products and strains belonging to different species and genera, commonly found in fermented foods, have been characterized for their decarboxylase activities.

It is known that this decarboxylase activity provides cell advantages because it allows increasing the environmental pH and leads to the energization of membrane. The genetic clusters responsible for BA production have been described individually and they can show differences, within the same amine, that depend mainly on the species and the strain. Nevertheless, it is interesting to note, that the decarboxylation mechanisms constitute an important ecological tool which can favor strain competitiveness in stressful conditions (i.e., acid stress and nutritional stress) [20,26,27].

Even if differences between the chromosomic decarboxylase clusters are present, some interesting consideration can be drawn. The first is that the presence of these cluster are usually strain and not species dependent and can be regarded as genomic islands, as demonstrated for TDC cluster in *L. brevis* by Coton and Coton [50]. In addition, the genes encoding different decarboxylase pathways in several LAB species (*L. saerimneri*, *L. brevis*, and *O. oeni*) are clustered on the chromosome, acting as a genetic hotspot related to acid stress resistance [19,20,192].

Although the knowledge concerning the origin and factors involved in BA production in fermented foods is well documented, it is difficult to prevent the accumulation of these compounds since the fermentation conditions cannot be easily modified and the aminobiogenic ability is strain dependent. For these reasons, the selection of specific LAB starters lacking the pathways for BA accumulation and able to outgrow autochthonous microbiota under production conditions is essential to obtain high quality food with reduced contents of these toxic compounds. In fact, the inability of a strain to synthesize BAs has to be included as a selective criterion for starter cultures [226]. On the other hand, the metabolic heterogeneity observed in natural starter cultures could open a serious concern about the presence of aminobiogenic LAB strains. This risk could be avoided with the use of defined starters or selected autochthonous strain mixtures, chosen based on the absence of such activity and endowed with taylor-made metabolic and functional features for specific products. Nevertheless, when undefined cultures need to be used, strategies to prevent the presence and growth of aminobiogenic LAB should be actuated. Among them, the use of food microorganisms able to degrade BAs previously synthesized in the food matrix should be taken into consideration.

## Figures and Tables

**Table 1:** Biogenic amines, precursors, decarboxylase enzyme and their producers lactic acid bacteria found in fermented foods.

Biogenic Amine	Amino Acid Precursor	Classification	Decarboxylase Enzyme or Pathway in LAB	Lactic Acid Bacteria Producing Species	References
Histamine	Histidine	Heterocyclic	Histidine decarboxylase (HDC)	*E. faecium, E. faecalis, L. sakei, L. curvatus, L. parabuchneri, L. buchneri, L. plantarum, L. brevis, L. casei, L. paracasei, L. vaginalis, L. reuteri, L. hilgardii, L. mali, L. rhamnosus, L. paracollinoides, L. rossiae, L. helveticus, S. thermophilus, O. oeni, P. parvulus, Leuc. mesenteroides, W. cibaria, W. confusa, W. paramesenteroides, T. muriaticus, T. halophilus*	[24,31,32,33,34,35,36,37,38,39,40,41,42,43,44,45,46]
Tyramine	Tyrosine	Aromatic	Tyrosine decarboxylase (TDC)	*E. faecium, E. faecalis, E. durans, E. hirae, E. casseliflavus, E. mundtii, L. sakei, L. curvatus, L. plantarum, L. brevis, L: buchneri, L. casei, L. paracasei, L. reuteri, L. hilgardii, L. homohiochii, L. delbrueckii* subsp. *bulgaricus, S. thermophilus, S. macedonicus, Lc. lactis, Leuc. mesenteroides, W. cibaria, W. confusa, W. paramesenteroides, W. viridescens, C. divergens, C. maltaromaticum, C. galliranum, T. halophilus, Sporolactobacillus* sp.	[9,17,32,47,48,49,50,51,52,53,54,55,56,57,58]
2-phenylethylamine	Phenylalanine	Aromatic	Tyrosine decarboxylase (TDC)	*E. faecium, E. faecalis, E. durans, E. hirae, E. casseliflavus, E. mundtii, L. brevis, Lc. lactis, Leuc. mesenteroides, C. divergens*	[9,15,16,17,32,42,47,59]
Cadaverine	Lysine	Aliphatic	Lysine decarboxylase (LDC)	*E. faecium, E. faecalis, L. curvatus, L. brevis, L. casei, L. paracasei, S. thermophilus, Pediococcus* spp., *Leuc. mesenteroides, T. halophilus*	[32,47,56,60,61,62,63]
Putrescine	Arginine	Aliphatic	Ornithine decarboxylase (ODC)	*E. faecium, E. faecalis, E. durans, E. hirae, E. casseliflavus, L. sakei, L. curvatus, L. buchneri, L. plantarum, L. brevis, L. paracasei, L. mali, L. rhamnosus, L. rossiae, L. homohiochii, Lc. lactis, S. thermophilus, S. mutans, P. parvolus, O. oeni, T. halophilus*	[20,30,32,47,54,56,60,64,65,66,67,68,69,70,71,72]
	Agmatine	Aliphatic	Agmatine deiminase (AgDI)	*E. faecalis, E. faecium, E. durans, E. hirae, E. mundtii, L. curvatus, L. plantarum, L. brevis, S. thermophilus, S. mutans, Lc. lactis, O. oeni, P. parvulus, P. pentosaceus, Leuc. mesenteroides, W. halotolerans, C. divergens, C. maltaromaticum, C. gallinarum*	
